# Supplementary figures and images for: Smell-induced gamma oscillations in human olfactory cortex are required for accurate perception of odor identity
Source: PLoS Biol. 2022 Jan 5;20(1):e3001509. doi: 10.1371/journal.pbio.3001509 (PMC8765613; doi:10.1371/journal.pbio.3001509)

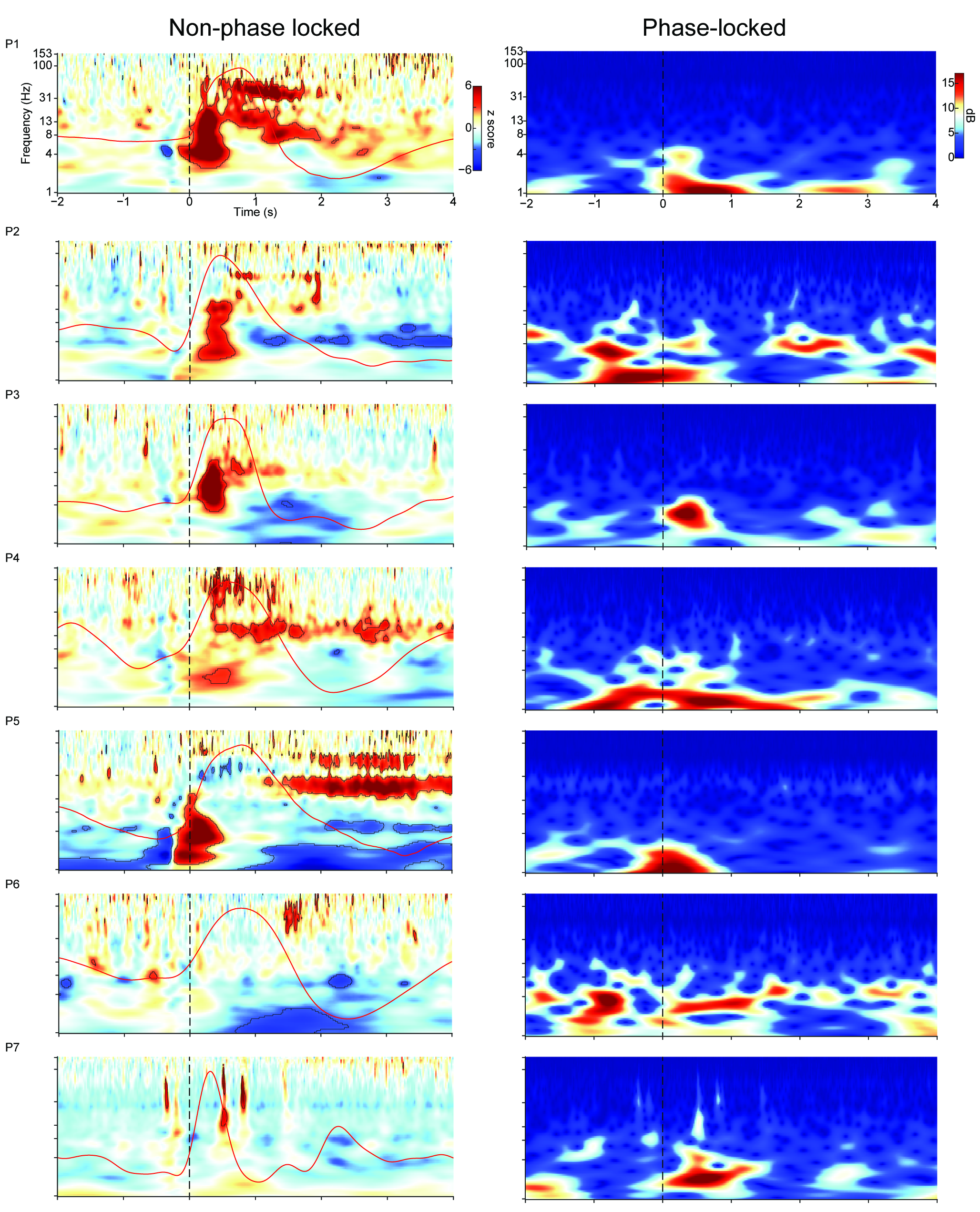

Supplement: S1 Fig — Odor-induced and odor-evoked spectrograms. Nonphase-locked (left) and phase-locked (right) spectrograms are shown for each participant (P1–P7). The nonphase-locked spectrogram was obtained by subtracting the event-related potential from each trial before time-frequency decomposition. The red solid overlay indicates each participant’s respiratory signal. Black outlines indicate statistically significant clusters (P < 0.05, FDR corrected). The phase-locked spectrogram was calculated as the baseline-corrected time-frequency decomposition of the event-related potential. The vertical short-dashed lines indicate sniff onset. The source is available in S5 Data. FDR, false discovery rate. (TIF) [file pbio.3001509.s001.tif]

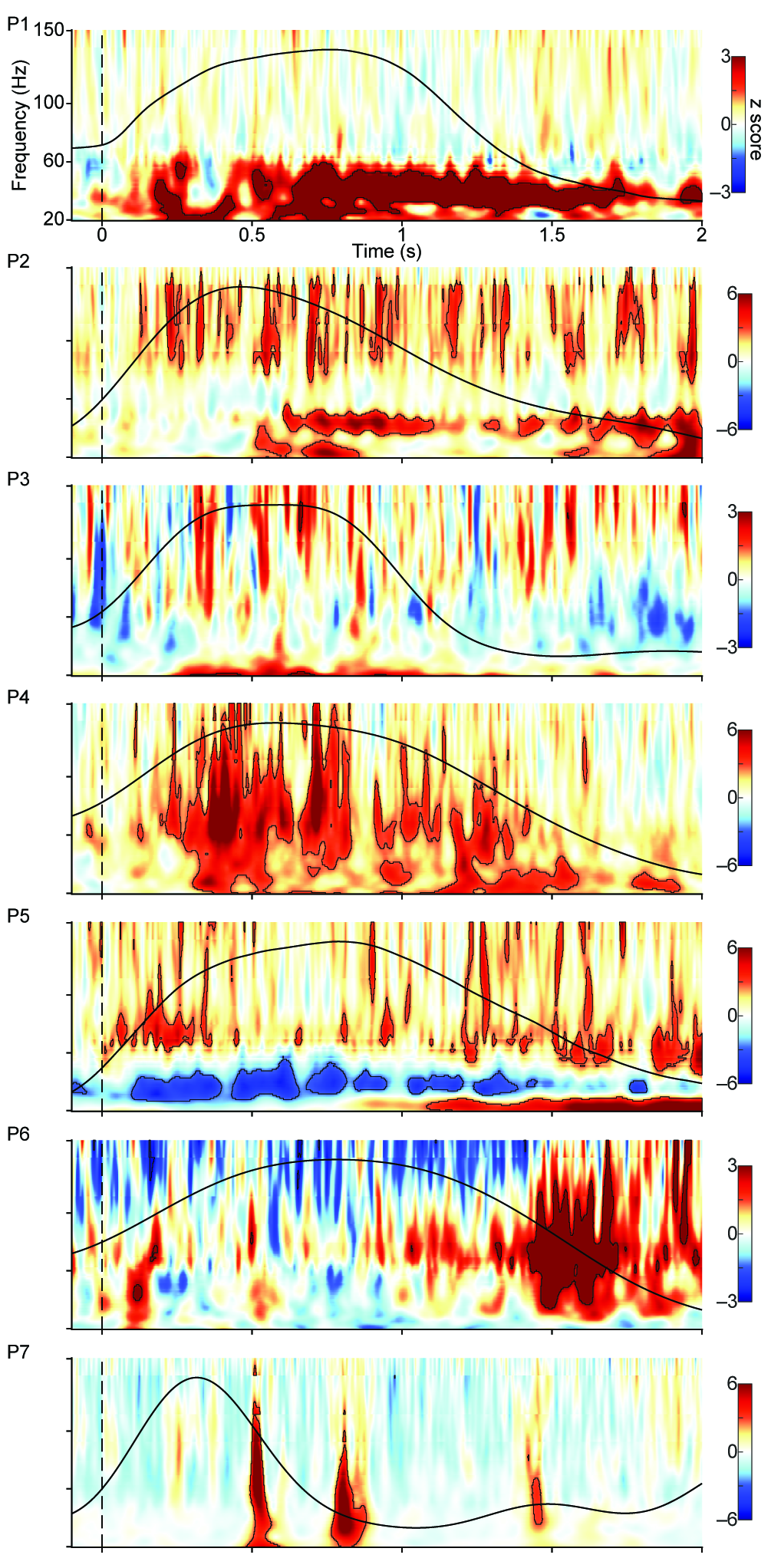

Supplement: S2 Fig — Sniff onset–aligned spectrograms with linear frequency scale. The black solid overlay indicates each participant’s (P1–P7) respiratory signal. Black outlines indicate statistically significant clusters (P < 0.05, FDR corrected). The vertical short-dashed line indicates sniff onset. The source is available in S6 Data. FDR, false discovery rate. (TIF) [file pbio.3001509.s002.tif]

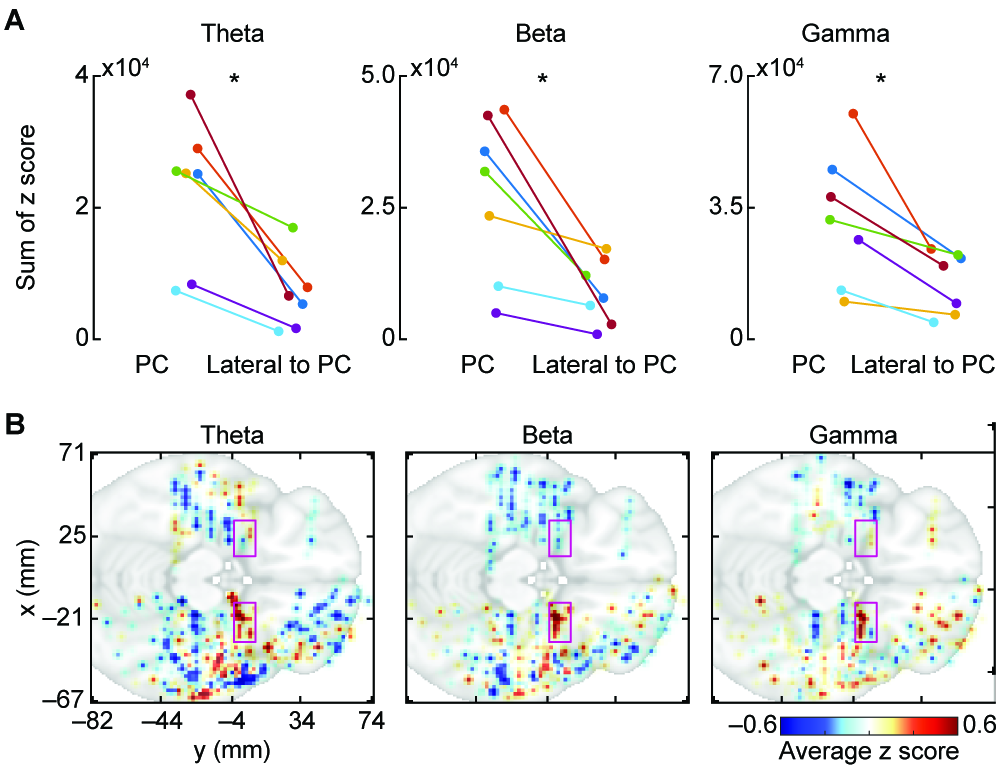

Supplement: S3 Fig — Odor-induced responses are maximal in PC. (A) Odor-induced amplitude (sum of z score) is larger in the depth wire located in the PC compared to those located outside of the PC (Lateral to PC) in theta, beta, and gamma frequency bands. * indicates statistically significant difference (two-tailed paired t test). (B) The mean z score was calculated over a time window of 2 s in the theta, beta, or gamma frequency band for each electrode and each participant. The data were collapsed over the z-axis and smoothed. The background brain is a slice (z = −16) of the MNI standard brain. The pink rectangles outline PC. The source is available in S6 Data. MNI, Montreal Neurological Institute; PC, piriform cortex. (TIF) [file pbio.3001509.s003.tif]

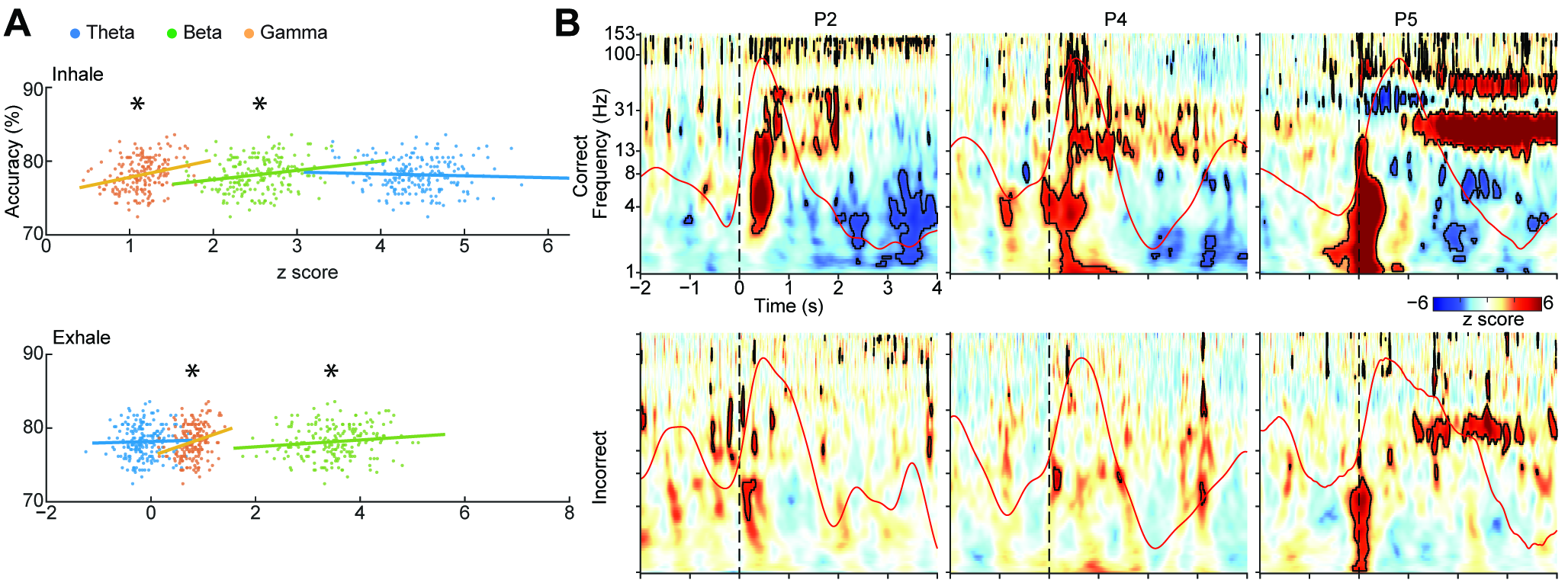

Supplement: S4 Fig — Odor-induced responses for correct and incorrect trials. (A) Example scatter plots showing the correlations plotted in Fig 4C. The scatter plot corresponding to the correlation value of 1 dot from each bar in Fig 4C is shown. (B) Representative spectrograms from 3 participants, showing correct (top) and incorrect trials (bottom) separately. The red solid overlay indicates each participant’s (P1–P7) respiratory signal. Black outlines indicate statistically significant clusters (P < 0.05, FDR corrected). The vertical short-dashed line indicates sniff onset. The source is available in S7 Data. FDR, false discovery rate. (TIF) [file pbio.3001509.s004.tif]

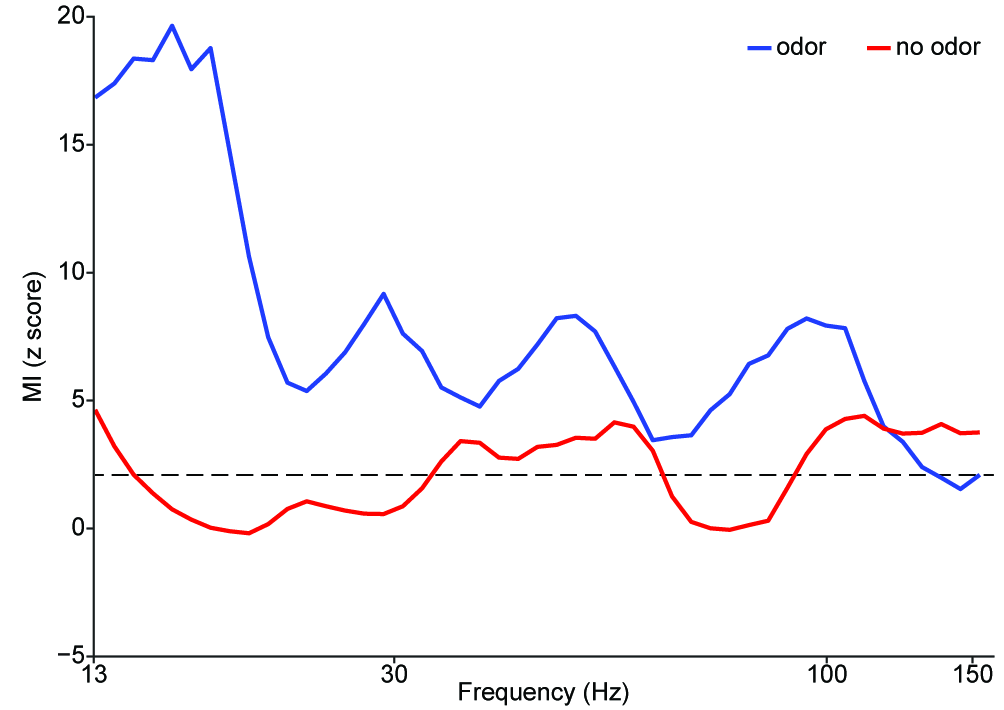

Supplement: S5 Fig — Results of MI computed for odor and no-odor trials, accounting for possible contribution of steep slope of sensory-evoked potentials (see Supplementary methods). The source is available in S7 Data. MI, modulation index. (TIF) [file pbio.3001509.s005.tif]

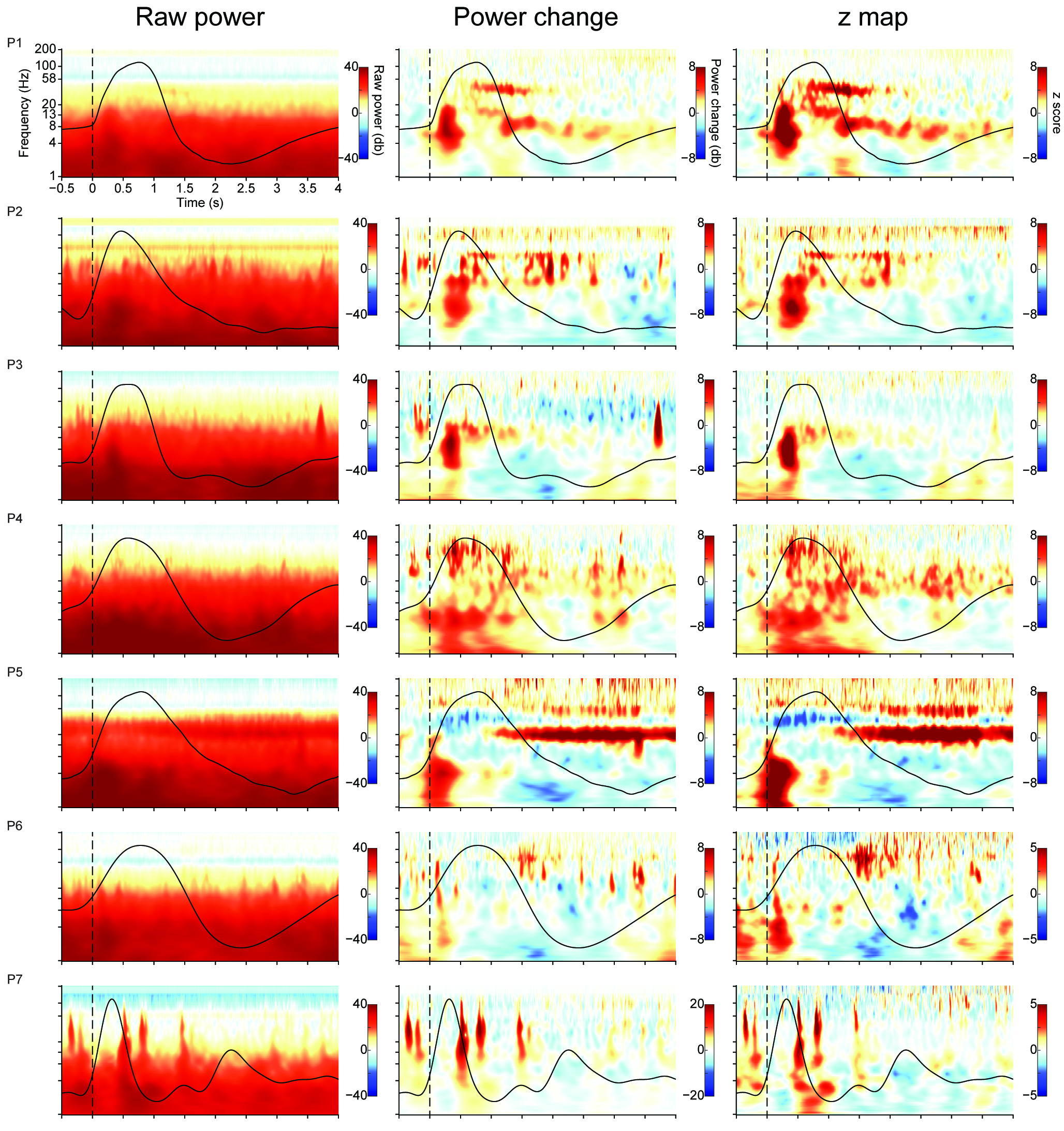

Supplement: S6 Fig — Sniff onset–aligned raw power (left), power change relative to baseline (middle), and z score map (right, same as Fig 2C) are shown for each participant (P1–P7). The baseline was defined as [−0.55, −0.05] s prior to cue onset. The black overlaid line indicates the respiratory signal. The vertical short-dashed line indicates sniff onset. The source is available in S8 Data. (TIF) [file pbio.3001509.s006.tif]
