# Supplementary material for: Smell-induced gamma oscillations in human olfactory cortex are required for accurate perception of odor identity
Source: PLoS Biol. 2022 Jan 5;20(1):e3001509. doi: 10.1371/journal.pbio.3001509 (PMC8765613; doi:10.1371/journal.pbio.3001509)
Supplement: S1 Text — (PDF) [file pbio.3001509.s007.pdf]

S1 Text. Supplementary methods

PC vs. lateral PC comparison

To examine the sniff-induced response in electrodes neighboring the piriform cortex contacts, we calculated the sniff-onset-aligned spectrogram for an electrode that was on the same wire as, but two contacts away from, the piriform cortex contact. Responses were quantified as the sum of z-scored amplitude values for each frequency band for each electrode, and results were compared using two-tailed paired, two-sample t tests.

Spatial distribution of theta/beta/gamma response

To examine the spatial distribution of the odor-induced spectrogram, we calculated the sniff onset-aligned spectrogram for each electrode and each patient. Then, the average z score across a time window of 2 s after sniff onset was calculated for theta (4–8 Hz), beta (13–30 Hz), and gamma (30–150 Hz) band separately. A 2-dimensional map of the spatial distribution of the sniff-induced response was created by collapsing the coordinates of all electrodes from all participants over the z-axis. The final map was smoothed using a 2-D Gaussian smoothing kernel with standard deviation of 0.75 (MATLAB's *imgaussfilt* function).

Linear scale spectrograms

To examine the gamma range of the spectrogram as reported in Fig 2 in the main text, we re-calculated the spectrogram using a linear frequency scale. We band-pass filtered

the raw LFP signals from 2 to 150 Hz, in steps of 1 Hz (bandwidth increase linearly from 2 to 30 Hz). Then, sniff-onset-aligned z-score maps were calculated using all trials for each participant using the method described in the main methods section, *Time-frequency analysis,* in the main text. Multiple comparisons were corrected using FDR method.

Phase-locked and non-phase-locked spectrograms

To dissociate non-phase-locked and phase-locked spectrograms, we removed the event-related potential (ERP) from single trials and re-calculated the spectrogram [1]. We segmented the raw LFP time series relative to sniff onset ([–5, 7] s). Note that the time window of interest is [–2, 4] s relative to sniff onset, which matches that in Fig. 2 in the main text. The extra data in the data epoching is to account for edge effects of band-pass filtering as described below. The ERP was calculated by averaging over all trials for each participant. To remove the ERP component from the raw data, the ERP was subtracted from each single trial for each patient. Time-frequency decomposition was performed for each trial using band-pass filtering and the Hilbert method, resulting in a time x frequency x trial amplitude matrix for each participant. We used 100 logarithmically spaced frequencies between 1 and 200 Hz, with the bandwidth logarithmically increasing from 2 to 50 Hz. The time-frequency decomposition was performed for the ERP time series using the same method and parameters.

To test the significance of the non-phase-locked spectrograms, we used a bootstrapping method, which differs from the method used in the main text because of

the difference in data organization. The spectrogram was first averaged over all trials and baseline-corrected by subtracting the average amplitude over the time window of [–0.55, –0.05] s relative to sniff onset. In each bootstrap, we randomly circularly shifted a random amount for each trial and calculated the baseline-corrected spectrogram. After repeating this procedure 1000 times, we obtained a null distribution of spectrograms at each time-frequency point. Then, the amplitude of the real spectrogram at each time-frequency point was transformed to z score by subtracting the mean of the null distribution, which was further divided by the standard deviation of the distribution. The mean and stand deviation of the null distribution was calculated using MATLAB's *normfit* function.

To visualize the spectrogram of the ERP time series, we converted the amplitude of the spectrogram into decibels (dB) of power. The resulting dB map was baseline corrected by subtracting the mean activity of the baseline ([–0.55, –0.05] s relative to sniff onset).

PAC control

To control for potential effects of sensory-evoked potentials on the MI results, we shuffled the trial-by-trial relationship between theta phase and higher frequency amplitude to test whether the modulation effect was due to the exact trial-by-trial relationship or rather induced by a steep slope during each trial. We followed the methods in Voytek et al. [2] exactly. We normalized the observed MI to the trial-order-shuffled null distribution and still found significant effects for the odor condition but not no-odor condition.

References

1. Cohen MX. Differences among total, phase-locked, and non-phase-locked power and intertrial phase consistency. Analyzing neural time series data. Massachusetts Institute of Technology; 2014. pp. 259–262.
2. Voytek B, D'Esposito M, Crone N, Knight RT. A method for event-related phase/amplitude coupling. Neuroimage. 2013;64: 416–424. doi:10.1016/j.neuroimage.2012.09.023
